# Seismomorphogenesis: a novel approach to acclimatization of tissue culture regenerated plants

**DOI:** 10.1007/s13205-013-0191-8

**Published:** 2013-12-25

**Authors:** Mostafa Khoshhal Sarmast, Hassan Salehi, Morteza Khosh-Khui

**Affiliations:** 1 Department of Horticultural Science, College of Agriculture, Shiraz University, Shiraz, Iran; 2 Present Address: Department of Plant Biology, University of California, Davis, CA 95616 USA

**Keywords:** Acclimatization, Shaking, *Sansevieria trifasciata* L., Thigmomorphogenesis, Tissue culture

## Abstract

Plantlets under in vitro conditions transferred to ex vivo conditions are exposed to biotic and abiotic stresses. Furthermore, in vitro regenerated plants are typically frail and sometimes difficult to handle subsequently increasing their risk to damage and disease; hence acclimatization of these plantlets is the most important step in tissue culture techniques. An experiment was conducted under in vitro conditions to study the effects of shaking duration (twice daily at 6:00 a.m. and 9:00 p.m. for 2, 4, 8, and 16 min at 250 rpm for 14 days) on *Sansevieria trifasciata* L. as a model plant. Results showed that shaking improved handling, total plant height, and leaf characteristics of the model plant. Forty-eight hours after 14 days of shaking treatments with increasing shaking time, leaf length decreased but proline content of leaf increased. However, 6 months after starting the experiment different results were observed. In explants that received 16 min of shaking treatment, leaf length and area and photosynthesis rate were increased compared with control plantlets. Six months after starting the experiment, control plantlets had 12.5 % mortality; however, no mortality was observed in other treated explants. The results demonstrated that shaking improved the explants’ root length and number and as a simple, cost-effective, and non-chemical novel approach may be substituted for other prevalent acclimatization techniques used for tissue culture regenerated plantlets. Further studies with sensitive plants are needed to establish this hypothesis.

## Introduction

In nature, plants as sessile organisms must respond to stimuli throughout their lifecycle. Mechanical conditioning is a physical stimulation or stress, deliberately applied, to manage plant growth and quality (Latimer 1991). Plant growth responses to tactile or contact stimuli have been termed thigmomorphogenesis (Jaffe 1973), while responses to shaking or vibrational stimuli have been termed seismomorphogenesis (Jaffe 1973). Paul-Victor and Rowe (2011) exposed *Arabidopsis thaliana* plants to brushing and three-point bending treatment. Their research results demonstrate that delayed development of key primary developmental features of the stem results in a ‘short and flexible’ rather than ‘short and rigid’ strategy for maintaining upright axes under mechanical stress; these changes correlate to major changes in tissue geometry-size and position of the pith, lignified interfascicular tissue and cortex as well as a reduction in density of lignified cells. Braam (2005) reviewed the plant responses to mechanical stimuli and has shown that the most common features of shoot thigmomorphogenesis among many different plant species are decrease in elongation growth and an increase in radial expansion and provides evidence in molecular level for how or why shaken plants become dwarves. Joshi et al. (2006) reported that in vitro acclimatization was more successful than ex vitro acclimatization as it provides a sufficient period for gradual exposure of plantlets to external environment. In tissue culture vessels, plantlet growth and development can be severely influenced by high relative humidity (RH) and media water potential. Plants produced under in vitro conditions when being transferred to ex vitro conditions are exposed to biotic and abiotic stresses and the major limitation in tissue culturing of plants is high mortality of plants transferred to field and greenhouse. To overcome these stresses and increase survival, plants need to acclimatize gradually because the plantlets produced under in vitro condition are often constrained by low space, low irradiance, high RH, large doses of growth regulators, high carbohydrate levels, and improper gas exchange. These situations may cause malformed stomata and poor epicuticular wax (Joshi et al. 2006). Mechanical stimulation is, in principle, an excellent means of limiting undesirable stem elongation, and it also can increase stem strength and specific chlorophyll content (Latimer 1991). Colon-Guasp et al. (1996) showed that there is no physiological advantage that would facilitate ex vitro hardening. The immediate transmission of Ca^2+^ and its accumulation in cytosol after thigmomorphogenesis treatments have led researchers to propose that Ca^2+^ may play a major role in signal transduction and after that this hypothesis was confirmed by experiments carried out on *Arabidopsis thaliana* which showed that after thigmomorphogenesis touch (TCH) genes encode calmodulin (CaM) and calmodulin like (CML) proteins (Chehab et al. 2009); however, four *TCH* genes in *Arabidopsis* had no counterparts with their orthologs in *Carica papaya* (Porter et al. 2009). In touch-stimulated *Arabidopsis* plants many genes involving in calcium-binding, cell wall modifying, defense, transcription factor, and kinase proteins up-regulated (Braam 2005). To our knowledge there has been no report on the using mechanical stress for adaptation of in vitro regenerated plantlets. The objective of the present study was to gain knowledge in the application of shaking treatment to acclimatize the tissue culture-regenerated plantlets of *Sansevieria trifasciata* L. as a model plant.

## Materials and methods

### Mechanical stress treatment

Tissue culture of snake plant (*Sansevieria trifasciata* L.) was performed according to the technique reported by Sarmast et al. (2009). Uniformly rooted explants were planted on half-strength MS medium (Murashige and Skoog 1962), in the jar glasses of the same size (nearly 3.6 cm ± 2 mm in length). Uniform plantlets were selected and used for shaking treatments. Explants were shaken in a horizontal plane using a shaker (Peco. Pooya Electronic, Iran). Explants in culture vessels, within a given experiment were treated twice daily at 6:00 a.m. and 9:00 p.m. for durations of 0 (control), 2, 4, 8, and 16 min at 250 rpm for 14 days. After applying the treatments, roots of explants were washed to remove agar and then transferred to a soil mixture (perlite:peat:loamy soil, with the same volume) and maintained in greenhouse under natural light (800 μmol m^−2^ s^−1^) at a day temperature of 27 ± 5 °C and RH of about 55 ± 5 %. In the first 5 days after transplanting, to increase RH, plantlets were covered with a sheet of light plastic (polyethylene sheet).

### Measurements

Forty-eight hours after transplanting, leaf length, and chlorophyll and proline content of treated plants were measured, and 6 months later mean leave length, leaf area, leaf diameter, chlorophyll content, substomatal CO_2_, CO_2_ reference, photosynthetic rate, PAR incident on leaf surface, and proline content were recorded. Photosynthetic rate (A_net_, μmol m^−2^ s^−1^), substomatal CO_2_ (Ci), CO_2_ reference (C_ref_), and PAR incident on leaf surface (Q_leaf_) were measured by portable photosynthesis meter (Lci, ADC, UK). Measurements were performed on clear sunny days between 10:00 a.m. to 1:00 p.m. (time of highest photosynthetic rate). Leaf area was measured by a leaf area meter (Delta-T. Devices Ltd), and the means of leaf area in each replication were used for analysis. Chlorophyll content was determined by spectrophotometer (SP 3000 Plus. Optica, Japan), (Saini et al. 2001) and proline measured using the method of Bates et al. (1973). Each experiment was carried out as a completely randomized design with five treatments and eight replications and each replication contained three explants. Data were analyzed using one-way ANOVA. LSD (*P* ≤ 0.05) of significance was applied to separate treatment means. The statistical analysis was done using SPSS version 16.0 (SPSS Inc., Chicago IL, USA).

## Results

### Forty-eight hours after shaking treatment

Several variables of snake plant leaves such as leaf length and chlorophyll content decreased but proline content increased after shaking treatments albeit no significant differences were found as compared with the control (Table 1). Sixteen-minute shaking treatment at 250 rpm decreased leaf length from 5.11 cm in control to 3.90 cm. Treatments of more than 8-min shaking at 250 rpm increased proline content in comparison with control plants. Root length and root number of explants increased after shaking treatments mainly in 8- and 16-min durations.

**Table 1:** Effects of shaking stress on leaf length, and chlorophyll and proline content of *Sansevieria trifasciata* L. plants (48 h after transplanting)

Treatment (250 rpm) (min)	Leave length (cm) (SD)^a^	Chlorophyll content (mg g^−1^ FW) (SD)	Proline content (μm g^−1^ FW) (SD)	Root number (SD)	Root length (SD)
0	5.11 a^b^ (0.47)	9.68 a (2.05)	29.32 ab (12.73)	4 b (1.00)	2.77 b (0.30)
2	5.57 a (0.67)	8.82 ab (1.05)	25.37 b (5.31)	5 ab (1.00)	3.45 b (1.36)
4	4.11 a (0.79)	6.37 c (1.28)	29.41 ab (1.73)	4 b (1.00)	3.67 b (0.57)
8	4.98 a (0.71)	7.56 a–c (0.40)	33.17 ab (2.55)	6 a (1.00)	5 ab (2.64)
16	3.90 a (0.31)	6.64 bc (0.58)	40.54 a (2.40)	5 ab (1.00)	6.91 a (1.69)

^a^*SD* standard deviation. ^b^ In each column, means with the same letters are not significantly different at ≤0.05 level of probability using LSD

### Six months after shaking

**Fig. 1:**
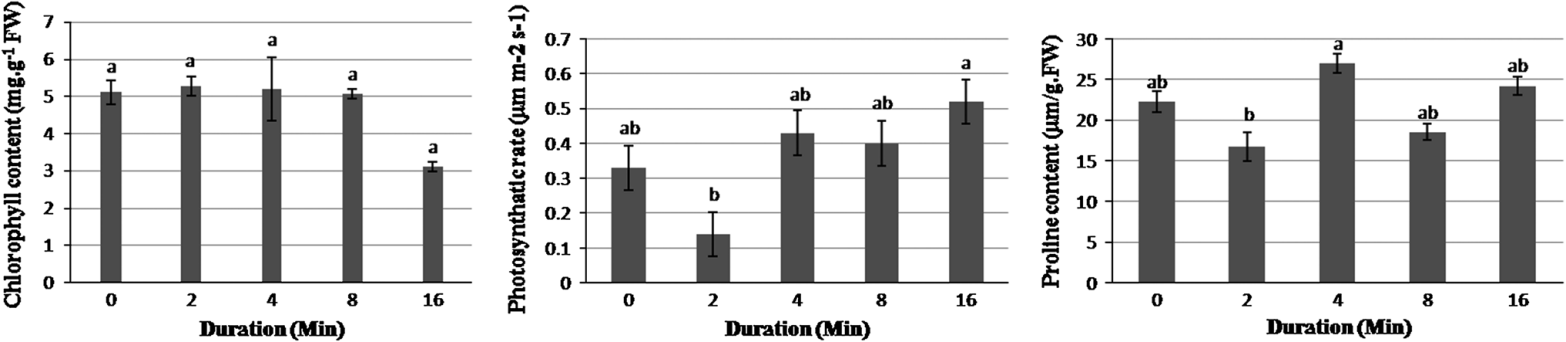
Effects of mechanical shaking on chlorophyll content (*left*), photosynthetic (*middle*) and proline content (*right*) of *Sansevieria trifasciata* L., 6 months after shaking. Data represent the mean ± SD

**Fig. 2:**
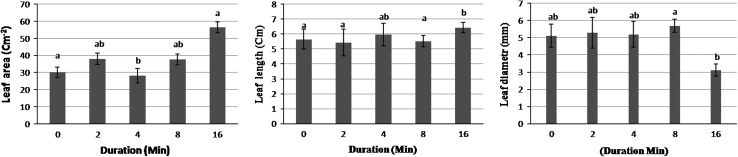
Effects of mechanical shaking on leaf area (*left*), leaf length (*middle*), and leaf diameter (*right*) of *Sansevieria trifasciata* L., 6 months after shaking. Data represent the mean ± SD

**Fig. 3:**
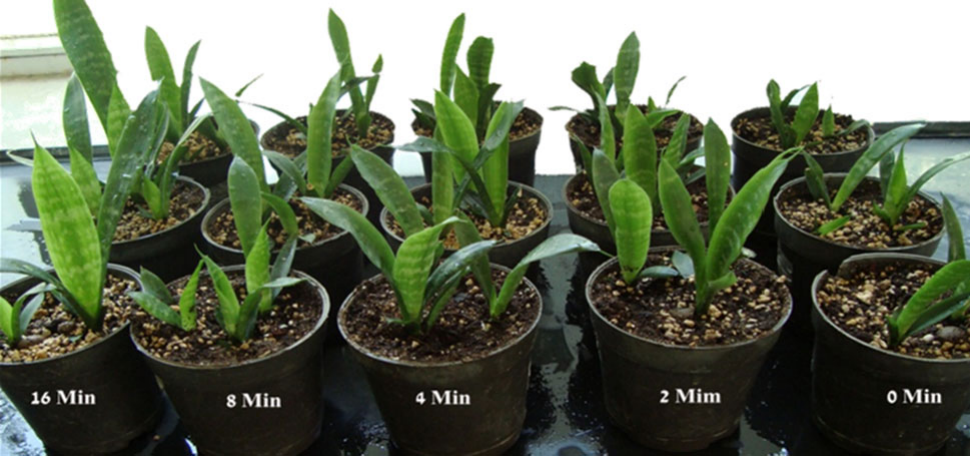
Thigmomorphogenesis of *Sansevieria trifasciata* L. plants, 6 months after shaking at 250 rpm for 14 days

Neither chlorophyll nor proline contents were significantly affected by shaking duration up to 8 min at 250 rpm after 6 months (Fig. 1), but in 16-min treatment, chlorophyll content of leaves decreased. However, 6 months later, following 16-min shaking treatment, leaf length and leaf area increased but leaf diameter decreased (Figs. 2, 3). Increase in photosynthetic rate to 0.51 μmol m^−2^ s^−1^ following 16-min shaking treatment was significantly higher than that following 2-min shaking treatment (Fig. 1). Control plantlets in relation to other shaken explants had a poor growth. Six months after starting the experiment, control plantlets had 12.5 % mortality; however, no mortality was observed in other treated explants.

**Fig. 4:**
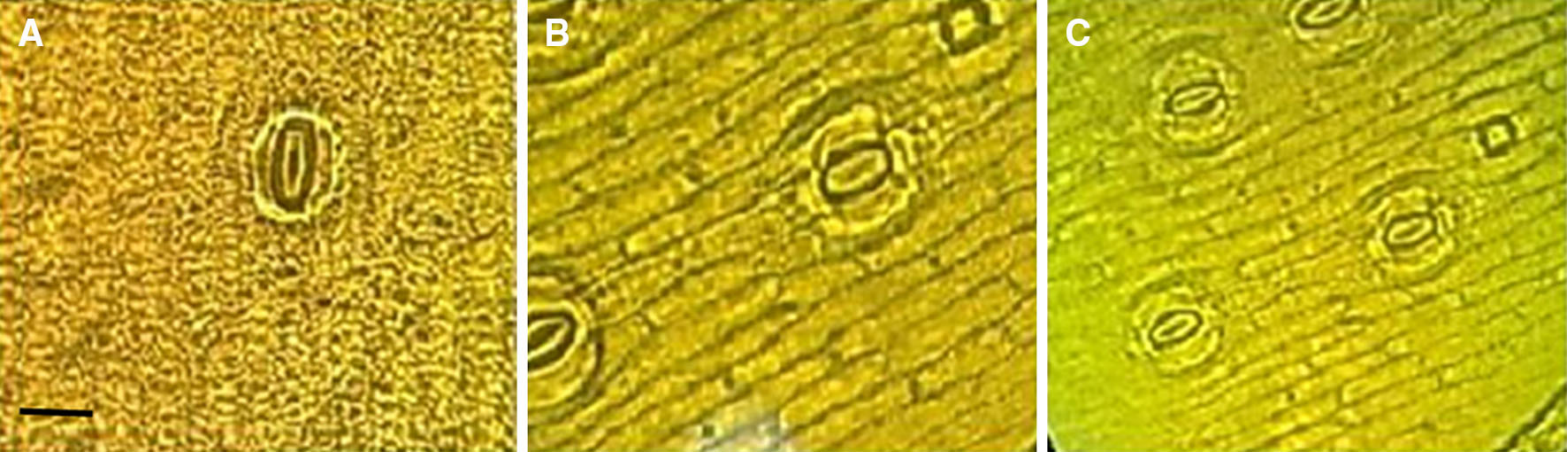
Stomatal situation of *Sansevieria trifasciata* L. leaf after shaking at 250 rpm. **a** Tissue cultured explant stomata 4 min after shaking at 250 rpm. **b** Control in vitro explants. **c** Acclimatized plant, 6 months after shaking. *Bar* 25 μm

Substomatal CO_2_ (C_i_), CO_2_ reference (C_ref_) and PAR incident on leaf surface (Q_leaf_) of *Sansevieria trifasciata* L. plants were not significantly affected by shaking treatments (Table 2) after 6 months. Figure 4 shows that control explants have wider stomata than shaken explants. Results indicated that some of control plantlets were lost after 6 months. Shaken explants had better morphological characteristics than control explants with expanded leaves. Leaves of untreated explants after transfer to greenhouse had been curled and finally lost. Just after shaking treatment leaf length of 16-min shaken explants decreased but 6 months later these plantlets had a highest length in relation to control. In fact results have shown that the preliminary stimuli prepare treated explants to withstand versus severe situation after transplantation.

**Table 2:** Effects of shaking stress on substomatal CO_2_ (C_i_), CO_2_ reference (C_ref_), and PAR incident on leaf surface (Q_leaf_) of *Sansevieria trifasciata* L. plants, 6 months after mechanical stress at 250 rpm

Treatment (min)	Cref (SD)^a^	C_i_ (SD)	Q_leaf_ (SD)
0	404.33 a^b^ (4.04)	393.00 ab (2.55)	71.66 a (5.77)
2	401.00 a (1.00)	348.00 ab (2.12)	68.66 a (2.04)
4	403.66 a (1.52)	197.33 b (8.30)	73.66 a (1.15)
8	403.00 a (2.64)	413.66 a (2.40)	69.66 a (2.88)
16	402.6 6 a (2.08)	314.00 ab (9.09)	73.33 a (3.51)

^a^*SD* standard deviation. ^b^ In each column, means with the same letters are not significantly different at ≤0.05 level of probability using LSD

## Discussion

The results of this experiment indicated the usefulness of mechanical stimulation as a means for better acclimatization of tissue culture regenerated plantlets. In a number of studies, using different types of stimulation resulted in increase in growth inhibition with increased mechanical stimulation in aster (*Callistephus chinensis* L.), dusty miller [*Senecio bicolor* (Willd.) Tod.], and petunia (*Petunia* spp.) (Autio et al. 1994), and chrysanthemum (Beyl and Mitchell 1977). Mechanical man-made stress improves the handling characteristics, appearance, and overall quality of vegetable transplants and some ornamental bedding plants (Latimer 1998). During hardening for ex vitro conditions, leaf thickness increased, while cuticle, epicuticular waxes, and stomatal density decreased (Pospisilova et al. 1999). Porter et al. (2009) have shown that *Carica papaya* seedling, mechanically perturbed by touch and water spry treatment, had higher lignin, dramatically less hypocotyl anthocyanins and chlorophyll, increased hypocotyl diameter, and decreased leaf width, stem length and root fresh weight related to control. Results of present study also revealed that leaf length decreased after treatments but adverse results were obtained after 6 months. The leaf length and area of *S. trifasciata* L. plantlets which received 16 min of shaking for 6 months increased. It is likely that shaking treatments improved morphophysiological characterization of the plantlets. Six months after starting the experiment, control plantlets had a 12.5 % mortality; however, no mortality was observed in other treated explants. Jaffe et al. (1984) and Latimer (1998) showed that percentage of cellulose in the fiber component of shaken plants can be increased to pass adverse environmental effects after transplanting. Furthermore, the plants were more responsive to mechanical stress in the morning than in the afternoon (Garner and Bjorkman 1996). In order to acclimatization of tissue culture regenerated plants, mechanical conditioning may be used as a substitute technique for plant growth regulators. Brushing transplant shoots is one of the most important mechanical conditioning treatments for vegetable transplants and ornamental bedding plants (Autio et al. 1994; Johjima et al. 1992; Latimer and Thomas 1991). Brushing provides a tactile stimulation on plants; hence, due to sterile condition in tissue culture, this procedure is inapplicable. Hence, shaking was applied without the abrasion associated with brushing. With increase of shaking time, root production was increased; this finding is similar to results presented by Garner (1994) on tomato’s (*Lycopersicum esculentum* L.) responses to wind. Long explants (nearly 3.7 cm) of snake plants were selected to impose shaking. Different treatments are being applied with growth retardants from the trizol group, such as paclobutrazol and ancymidol, gas exchange and CO_2_ enrichment, sugar-low and sugar-free medium, reduced humidity, and presence of root prior to transplanting, plug system in vitro, reducing MS salts or using media with lower levels of nitrogen, increased levels of agar, elevated irradiance, and liquid medium, for better acclimatization of plantlets which was reviewed (Ziv 1995). Reduced deposits of epicuticular waxes, the incapability of the plantlet stomata to close shortly after removal from culture, and deficient root system are the pivotal causes for water losses from leaf, desiccation, and poor survival (Fabbri et al. 1986; Sutter et al. 1992), and also in vitro adaptation should provide a microenvironment to develop leaf and root structure that can withstand the transpiration and support photosynthetic activity under stress conditions during the early phases of acclimatization ex vitro (Ziv 1995). In vitro to ex vitro transfer of plantlets caused increase of chlorophyll *a* and *b* contents (Rival et al. 1997). In the present experiment, treatment of 16 min shaking, probably due to increase of leaf surface, necessitates that amount of chlorophyll in an unit of leaf surface be less than that in other treatments. High photosynthetic rate in highest treatment level (16-min) had shown better set up rather than control treatment. Inhibition of stem elongation is correlated with disrupting of polar auxin transfer and release of additional ethylene in culture vessel due to limitation of space; ethylene content in culture medium may exceed a certain threshold (Mitchell 1996). It has been reported that GA (gibberellic acid)-like hormones will decrease after seismic treatment in tissue target (Mitchell 1996). We conjectured that 14 days immediately after conditioning treatments, levels of endogenous growth inhibitors such as ABA increased (Beyl and Mitchell 1983; Erner and Jaffe 1982); therefore, plants that received shaking treatment must be shorter than control. Additionally, at the cellular level it has been shown that seismic stress affects the cell wall by both decrease in cell expansion and increase in stem monolignol polymerization (Boyer et al. 1979). Peroxidase activity that is correlated with lignification will increase and inhibition of plant elongation will be observed (Saidi et al. 2009). Such conditioning treatments reduced growth and water status in short-term but did not reduce long-term growth (Latimer and Mitchell 1988). The same result was obtained in present experiment. Results indicated that shaking treatment increased root growth and proline content. The research results indicated that this method can be considered as a substitute technique for adaptation of tissue culture regenerated plants. In response to thigmomorphogenesis not only intracellular calcium, jasmonates, ethylene, abscisic acid, auxin, brassinosteroids, nitric oxide, and reactive oxygen species but also many genes implicated in various cellular processes such as calcium sensing, cell wall modifications, and defence have been affected (Chehab et al. 2009). Braam (2005) has been taken a molecular approach by *TCH* genes of *Arabidopsis* that are rapidly and strongly upregulated in expression in response to various environmental stimuli that encode calmodulin, calmodulin-related proteins, and a xyloglucan endotransglucosylase/hydrolase (XTH) predicted to act in modifying the plant cell wall and collaborate in the fundamental process of plant cell expansion (Braam 2005). The technique presented in this article may be applicable to a wide range of ornamental and other crops, but a thorough study, with different plants along with costs calculation, should be conducted to determine which approach serves better the purpose of acclimatization in vitro. Although many trials have been carried out to determine the mechanisms by which plants sense environmental stresses, transduce signals into cells, and regulate cellular and organismal alterations, it is clear that we have a very limited understanding of the these processes that this entire area needs enhanced research efforts.

## Abbreviations

ABA: Abscisic acid
BAP: Benzyl amino purine
CaM: Calmodulin
CML: Calmodulin like
GA: Gibberellic acid
IBA: Indole-3-butyric acid
RH: Relative humidity
TCH: Thigmomorphogenesis touch genes
